# Some Random Observations on Tuberculosis

**Published:** 1904-12

**Authors:** 


### Some Random Observations on Tuberculosis.

[*Medical Record*, October 29, 1904.]

A. A. Eshner presents a paper intended to emphasise the fact that tuberculosis is a transmissible disease, that it is a preventable disease, and that it is a curable disease. Strictly speaking, tuberculosis is not an inherited disease, and what is transmitted to the offspring is some vice of tissue, some aberration of function by reason of which a predisposition to tuberculosis is set up. Much is to be done in the safeguarding of children by proper attention to the cattle used as milk producers, and in restricting promiscuous expectoration and other means of spreading the disease. Typhoid fever and tuberculosis are sometimes difficult to differentiate, and they may also be concomitant, each disease sometimes occurring as a complication of the other. There is at times a close similarity between influenza and the symptoms of incipient tuberculosis or of exacerbations of the tuberculous process.

The prime indication in the treatment of a case of tuberculosis is the improvement of the general nutrition by whatever means possible. This will be met by an abundant supply of fresh air, good, assimilable, nutritious food, and a proper amount of exercise and rest. Two other requirements are essential in the management—namely, isolation and disinfection.

